# Right Parietal rTMS Induces Bidirectional Effects of Selective Attention upon Object Integration †

**DOI:** 10.3390/brainsci15050483

**Published:** 2025-05-03

**Authors:** Markus Conci, Leonie Nowack, Paul C. J. Taylor, Kathrin Finke, Hermann J. Müller

**Affiliations:** 1 Department of Psychology, Ludwig-Maximilians-Universität München, D-80802 Munich, Germany; 2 Munich Center for Neurosciences—Brain & Mind, Ludwig-Maximilians-Universität München, D-82152 Planegg-Martinsried, Germany; 3 Memory Center, Department of Neurology, Jena University Hospital, D-07747 Jena, Germany

**Keywords:** perceptual grouping, object integration, visual attention, visual extinction, rTMS, intraparietal sulcus, parietal cortex

## Abstract

**Background/Objectives:** Part-to-whole object completion and search guidance by salient, integrated objects has been proposed to require attentional resources, as shown by studies of neglect patients suffering from right-parietal brain damage. The current study was performed to provide further causal evidence for the link between attention and object integration. **Methods:** Healthy observers detected targets in the left and/or right hemifields, and these targets were in turn embedded in various Kanizsa-type configurations that systematically varied in the extent to which individual items could be integrated into a complete, whole object. Moreover, repetitive transcranial magnetic stimulation (rTMS) was applied over the right intraparietal sulcus (IPS) and compared to both active and passive baseline conditions. **Results:** The results showed that target detection was substantially facilitated when the to-be detected item(s) were fully embedded in a salient, grouped Kanizsa figure, either a unilateral triangle or a bilateral diamond. However, object groupings in one hemifield did not facilitate target detection to the same extent when there were bilateral targets, one inside the (triangle) grouping and the other outside of the grouped object. These results extend previous findings from neglect patients. Moreover, a subgroup of observers was found to be particularly sensitive to IPS stimulation, revealing neglect-like extinction behavior with the single-hemifield triangle groupings and bilateral targets. Conversely, a second subgroup showed the opposite effect, namely an overall, IPS-dependent improvement in performance. **Conclusions:** These explorative analyses show that the parietal cortex, in particular IPS, seems to modulate the processing of object groupings by up- and downregulating the deployment of attention to spatial regions were to-be-grouped items necessitate attentional resources for object completion.

## 1. Introduction

**Figure 1:**
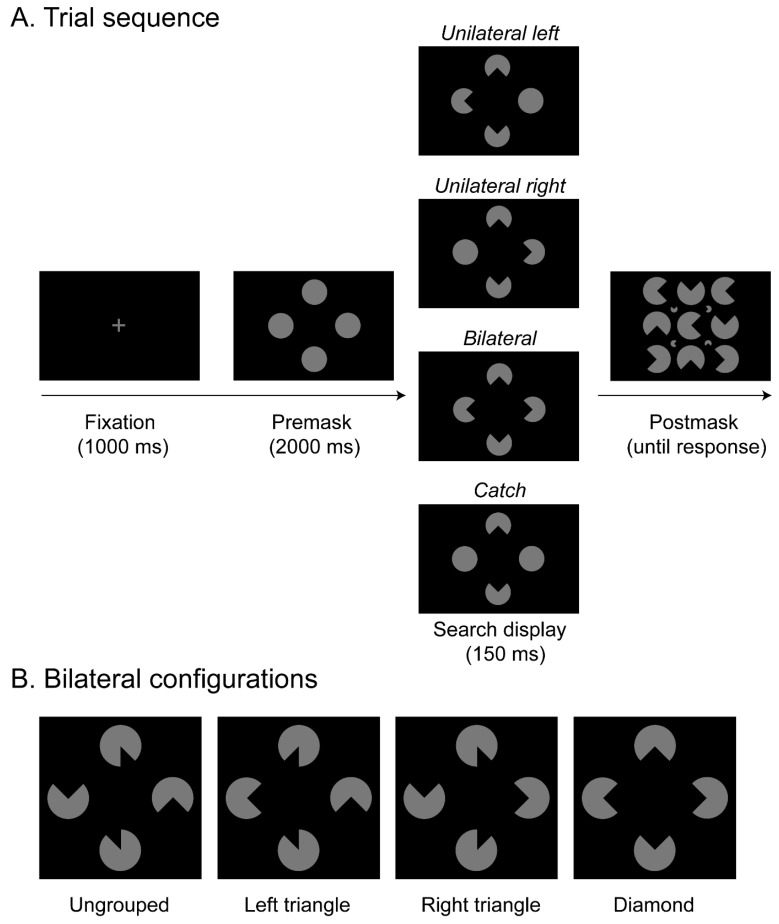
(**A**) Example trial sequence. First, a fixation cross was shown for 1000 ms, followed by a premask display presented for 2000 ms. Next, participants saw a Kanizsa-type configuration which was shown for 150 ms with quarter segments removed from the top and bottom, and from either the left side (unilateral left), the right side (unilateral right), both sides (bilateral), or no side (catch), as depicted in the example search displays from top to bottom, respectively. Finally, a postmask display (with nine big and four small disks arranged in random orientation) was presented until a response was given. In the example trial sequence, search displays present possible variants of a diamond configuration. (**B**) Examples of the four different types of object groupings presented in bilateral trials (i.e., displays containing target cut-out segments in both hemifields): In the diamond configuration, a complete illusory figure spanning across both hemifields was visible (right panel). The right triangle condition (middle-right panel) presented an illusory triangle confined to only the right hemifield, and in the left triangle condition (middle-left panel) an illusory triangle emerged in only the left hemifield. The ungrouped configuration (left panel), which did not lead to the emergence of any illusory figure, served as a baseline.

Perceptual grouping acts to structure cluttered input from the visual environment, by integrating fragmentary visual information into coherent whole objects. One famous example that illustrates such object integration processes is the “Kanizsa figure” ([1]; see Figure 1), which depicts several aligned “pacmen” inducer elements that are grouped, thereby leading to the emergence of an illusory figure (e.g., a diamond or triangle) while lacking a corresponding physical object. Kanizsa figures, thus, demonstrate the capability of the visual system to generate coherent wholes from fragmentary parts.

Prominent theories, such as the “feature integration theory” [2] in turn postulated that object integration arises from higher-level cognitive processes that depend on the allocation of selective attention. Conversely, several studies suggested that object integration is achieved preattentively, that is, prior to the engagement of attention, thus, supporting accounts of object-based attention (see ref. [3]). A common approach to explore the relationship between selective attention and object integration is to test neurological patients with brain lesions in the right inferior parietal cortex, which often results in associated spatial attention deficits. Such selective impairments frequently lead to a condition of hemispatial neglect and associated extinction behavior [4,5], which manifests in a failure to orient towards stimuli presented in the contralesional hemifield. However, despite severe inattention to one part of the visual field, these patients often show preserved access to integrated object information [6]. For instance, Mattingley and colleagues [7] (see also ref. [8]) presented search displays with either Kanizsa-type or comparable ungrouped configurations to an extinction patient and asked her to detect the removal of segments from circular disks in the left and/or right hemifield. She was able to detect unilateral target offsets on both sides. However, in ungrouped configurations, severe extinction behavior emerged when the segments were removed from both sides. In this case, the patient missed the left-sided targets and only reported the right-sided targets. However, when the cutout segments were arranged such that they could be grouped together to form a coherent whole object across both hemifields, extinction behavior was substantially reduced, thus showing that the patient had access to the grouped object despite severe (left-sided) inattention. This finding was, thus, taken to suggest that object integration occurs preattentively.

In contrast to these findings that would support a preattentive integration account, several studies also provided support for a crucial role of attention during object integration [9,10,11]. For instance, the study by Nowack et al. [9] tested a sample of neglect patients in a visual search task that again involved the detection of targets in the left and right hemifields. Search displays provided different configurations of Kanizsa figures that varied in their extent of perceptual grouping (Figure 1). Critically, in that study, the grouped objects were systematically varied and either only occurred in the left or right hemifield (e.g., presenting a Kanizsa triangle), or the grouping expanded across both hemifields (thus revealing a Kanizsa diamond; see Figure 1B). The results showed that when individual target segments were not grouped across hemifields, detection was compromised, thus revealing extinction, as opposed to a substantially improved detection performance with a bilaterally grouped diamond configuration (as shown previously in various other studies, see, e.g., [7,8,10,12] for a review). Moreover, a target within a salient Kanizsa triangle presented in the attended, right hemisphere was readily detected. Likewise, the detection of a target in a salient triangle presented in the unattended, left hemifield was also rather good. The very same triangle, however, completely failed to improve contralesional target detection whenever it was presented together with another ipsilesional and structurally non-integrated target. This was taken to suggest that attention was captured by the salient grouped object in the unattended, left hemifield only when it was not engaged in processing the isolated target in the attended, right hemifield. These findings, thus, extend previous studies and show that attentional spreading from the attended to the neglected hemifield is crucial for object integration to facilitate performance.

Studies with neurological patients provide only one source to decide between competing theories of object integration. Given that performance of brain-damaged patients might not be representative for normal processing [13,14], they should ideally be complemented by studies allowing for inferences in healthy observers to be drawn. In the current study, we, therefore, used repetitive transcranial magnetic stimulation (rTMS) to induce brief and reversible disruptions in spatial attention, thereby allowing us to assess the role of selective attention on perceptual grouping in normal brain function [15]. Several previous findings with TMS indeed suggest that the parietal cortex is relevant for spatial attentional functioning. For instance, a study by Hilgetag et al. [16] applied unilateral TMS over the right and left parietal cortex and observed extinction of a contralateral stimulus whenever it was presented together with a second, ipsilesional stimulus—comparable to the typical finding in neglect patients. The subject’s attention towards the ipsilesional stimuli, however, improved significantly. In general agreement with these findings, various other studies also showed that a disruption of the posterior parietal cortex can generate attentional deficits, which may be revealed by failures to detect [17,18,19,20,21] or to identify [22] targets in the visual field contralateral to the stimulation site and under conditions of bilateral simultaneous presentation [23] (see also [24,25], for reviews). Such attentional failures not only occur when objects need to be integrated in the visual modality, but similar effects can also be revealed with multimodal stimulation [26]. Moreover, it should be noted that parietal TMS stimulation not only induces neglect-like deficits in performance but may conversely also boost visual attention both in patients [27,28,29] and in healthy volunteers [16,30,31] (for a review see [32]). Moreover, it is commonly reported that theta-burst stimulation causes a high interindividual variability arising from variations in brain plasticity [33,34]. Together, these findings, thus, demonstrate a causal involvement of the parietal cortex in spatial attentional orienting, while the TMS stimulation may eventually induce both performance costs and benefits.

The current study was performed to further test the causal role of selective spatial attention for object integration and to extend previous findings reported with neglect patients to healthy observers. Accordingly, we made use of offline rTMS and stimulated the right intraparietal sulcus (IPS). In the experiment, a sample of healthy participants would be presented on a given trial (see Figure 1A) with a search display that contained four disks, and the task was to indicate whether segments were removed from the left disk, the right disk, from the disks on both sides, or not at all. Variations in the orientations of the removed segments in turn generated different variants of an illusory figure comparable to the stimulus configurations presented in Nowack et al. [9]: a whole Kanizsa “diamond” spreading across both hemifields and a Kanizsa “triangle” confined to only one hemifield. This allowed an assessment of whether parietal stimulation modulates target detection performance in the two visual hemifields (ipsi- and contralateral to the critical rTMS stimulation over area IPS). Importantly, since several studies reported that various forms of masking can substantially reduce the visibility of Kanizsa figures [35,36,37,38,39], we included a cluttered postmask after the presentation of the stimulus display in order to decrease the visibility of the target stimuli, hence making it a harder task for the healthy participants (for a review, see [40]). Each participant completed three experimental sessions that varied in terms of the type of TMS stimulation that was applied (IPS—experimental, M1—active baseline, no rTMS—passive baseline). We expected IPS transcranial magnetic stimulation to explicitly influence the selection of (grouped) objects.

## 2. Materials and Methods

### 2.1. Participants

A total of 17 right-handed participants (7 males, *M* = 25.7 years, *SD* = 3.9 years) with normal or corrected-to-normal vision took part in the experiment. Participants either received monetary compensation (10 Euros per hour) or course credits for taking part in the experiment. The experimental procedure was approved by the local ethics committee (Department of Psychology, Ludwig-Maximilians-University, Munich; protocol code: “29_Nowack_b”, date of approval: 16 November 2020), and written informed consent according to the Declaration of Helsinki was obtained from all participants.

Sample size was determined on the basis of an a priori power analysis. We aimed for 95% power to detect an *f*(*U*) effect size of 1.08 (partial *η*^2^ = 0.54) at an alpha level of 0.05 and a nonsphericity correction of 1. This effect size was based on a previous study, which used comparable stimuli and a variant of a detection task that also tested healthy observers [41]. A TMS-dependent modulation of attention and concurrent object integration processes in the current experiment would be reflected in a significant 3-way interaction [Configuration × Target × TMS-stimulation], which—according to our analyses—would require only 6 participants in a within-subjects design. However, effects of TMS upon visual processing and attention are typically rather varied across participants and previous studies, therefore, typically used larger sample sizes (e.g., [17,28,42]). Given this, we decided to test a larger sample size with a total of N = 17 participants (It should be noted that we initially tested N = 20 participants, but three participants had to be excluded from the data proper because they performed well below chance level in the (important) bilateral target displays in the IPS (*M* = 16.5%), the M1 (*M* = 11.5%), and the no rTMS (*M* = 16.9%) stimulation conditions (all other participants were much more accurate in responding to bilateral targets across the various stimulation conditions, *M* = 88.7%). Hence, the results reported here are based on a sample of 17 participants (which is still well above the minimal sample size as suggested by our power calculations reported above).

### 2.2. Apparatus and Stimuli

The experimental routine was programmed using the Psychophysics toolbox [43] in combination with Matlab [44]. The experiment was conducted in a sound-attenuated room that was dimly lit. During the experiment, the head of the participant was stabilized by a forehead and chin rest, positioned 57 cm from a 17-inch monitor (1024 × 768 pixels screen resolution, 85 Hz refresh rate). Eye movements were recorded from the right eye at a sampling rate of 250 Hz using an Eyelink CL eye tracker system (SR-Research Ltd., Kanata, ON, Canada). At the beginning of each block, a 5-dots calibration routine was performed. To ensure that observers remained fixated at the screen center, the eye gaze was monitored, and a given trial was discarded if participants moved their gaze more than 1.3° away from the central fixation cross, thus revealing an overt orienting response. This was the case in 9.6% of all trials.

Stimuli were the same as used in the study by Nowack et al. [9] and consisted of four gray disks (3.81 cd/m^2^), each subtending a diameter of 1° of visual angle. The stimuli were presented against a black background (0.01 cd/m^2^). The disks were arranged in diamond form subtending 3.5° × 3.5°, and their distance from the central fixation cross was 1.3°. Each trial started with the presentation of a premask display (with complete, circular placeholder disks), followed by a briefly presented search display where segments were cut out from the placeholders, thus revealing various Kanizsa-type stimulus configurations (see the Supplementary Figure S1 for all possible arrangements). Subsequent to the search display, a densely cluttered postmask display was presented that consisted of 9 large and 4 small disks with removed segments, depicting variable orientations of the cut-out parts. Note that this postmask only presented arrangements where the individual segments would not give rise to an illusory figure (see Figure 1A for an example of the postmask stimulus). There were four different types of search display: unilateral left displays consisted of two central disks (one above and one below fixation) and the disk to the left of fixation, which all had a segment cut out whereas the right disk was complete (i.e., without cut-out section); in unilateral right displays, segments were removed from the right and the central disks, and the left disk was complete. In bilateral displays, all four circles were presented with cut-out segments. Finally, in catch trials, only the central (i.e., the top and bottom) disks had cut-out sections, whereas the left and right disks were both complete. Note that catch trials were presented to obtain a measure for guessing. Examples of all four types of search display are depicted in Figure 1A.

For each of these search display types, four variants of object groupings were generated through systematic changes in the orientation and size of the cut-out segments (that is, at orientations of 0°, 90°, 180°, or 270°, and with a “pie” segment of 1/4 or 1/8 removed, respectively; see Figure 1B for examples of these types of object groupings in bilateral target displays and Supplementary Figure S1 for all possible arrangements). For the diamond configuration (Figure 1B, right), the segmented disks were arranged such that, in the bilateral condition, a complete Kanizsa-type illusory diamond emerged across both hemifields from the inward-facing indents in the disks [45]. In addition, two variants of this configuration presented a complete Kanizsa-type illusory triangle, either in the right hemifield (right triangle, Figure 1B, middle-right) or in the left hemifield (left triangle, Figure 1B, middle-left). Note that, in bilateral conditions, the cutout segment in the other hemifield was presented such that it did not integrate with the triangle, facing randomly either the top or bottom. Finally, ungrouped configurations were arranged pseudo-randomly such that no illusory figure emerged within the left or the right hemifield: the disks with missing quarter-segments on the left and/or right faced up and down, and the cut-out segments in the top and bottom disks faced to the left and right, respectively (see Figure 1B, left).

### 2.3. Procedure and Behavioral Task

The experimental procedure was adopted from the study by Nowack et al. [9]: Each trial started with the presentation of a fixation cross at the center of the screen for 1000 ms. This was followed by a premask display, which presented four complete disks in a diamond arrangement around fixation for 2000 ms. Next, the search display presented one of the four possible object configurations (see examples of bilateral displays in Figure 1B). In the search display, segments were removed from the top and the bottom and from either the left side, the right side, both sides, or from neither left nor right side (see Figure 1A). Thus, zero to two segments were removed from the left and right circles, and these served as the to-be-detected targets, whereas the two segments on the top and bottom were response-irrelevant distractors. The search display was presented for 150 ms. The optimal presentation time of the search display was determined prior to the main experiment in a separate pilot study, which tested a group of 11 participants and compared various presentation times with the aim to achieve an overall accuracy of around 80%. Subsequent to the search display, a postmask appeared, displaying 9 large and 4 small disks with removed segments, with variable orientations of the cut-out parts (note that the orientations of the segments in this postmask were arranged such that they would not induce an illusory figure or a grouped object). The postmask was shown until the participants indicated on which side(s) a target segment was removed from the search display via keyboard press (four response alternatives: left [key 1], right [key 2], both [key 3], or none [key 4]). Each trial was separated from the next by a blank screen (with central fixation cross), which was shown for 1000 ms. Figure 1A presents an example trial sequence and possible target types presented in the search displays, illustrating where the cut-out segments could be removed from a given configuration.

A given session of the experiment consisted of 288 experimental trials, which were presented in eight blocks of 36 trials each, with a break after each block. Each block consisted of 8 unilateral left, 8 unilateral right, 16 bilateral, and 4 catch trials, presented in a randomized order. The various types of object configuration (ungrouped, left triangle, right triangle, or diamond) were presented in randomized order across the whole experiment. Each participant completed three experimental sessions (on three separate testing days), where each session would be identical in terms of the experimental setup, except for the type of TMS stimulation that was applied (IPS—experimental, M1—active baseline, no rTMS—passive baseline, see further details below). All three experimental sessions (with each of the TMS stimulation conditions) were administered in counterbalanced order and participants were not told which condition was applied. In summary, the experiment varied three experimental factors: object configuration (ungrouped, left triangle, right triangle, or diamond), target (unilateral left, unilateral right, bilateral, catch), and TMS stimulation (IPS, M1, no rTMS).

### 2.4. Transcranial Magnetic Stimulation

We applied continuous theta burst rTMS triplets of pulses at 50 Hz (presented in bursts at 5 Hz, intensity = 80% active motor threshold, duration = 40 s, i.e., 600 pulses) by using a figure-8 coil (PowerMAG research 100 machine with a coil with an outer winding diameter of 95 mm, MAG & More GmbH, Munich, Germany). TMS was applied offline at the beginning of each of the three experimental sessions. In each session, the TMS stimulation would be either (i) applied to the target site (IPS), (ii) applied to an M1 control site (active baseline), or (iii) would not be applied (passive baseline, no rTMS). Coil positioning used a neuronavigation system via frameless infrared stereotactic registration (Brainsight, Rogue Research, Montreal, QC, Canada) to determine the stimulation sites based on the participant’s T1 weighted structural MRI scans.

**Figure 2:**
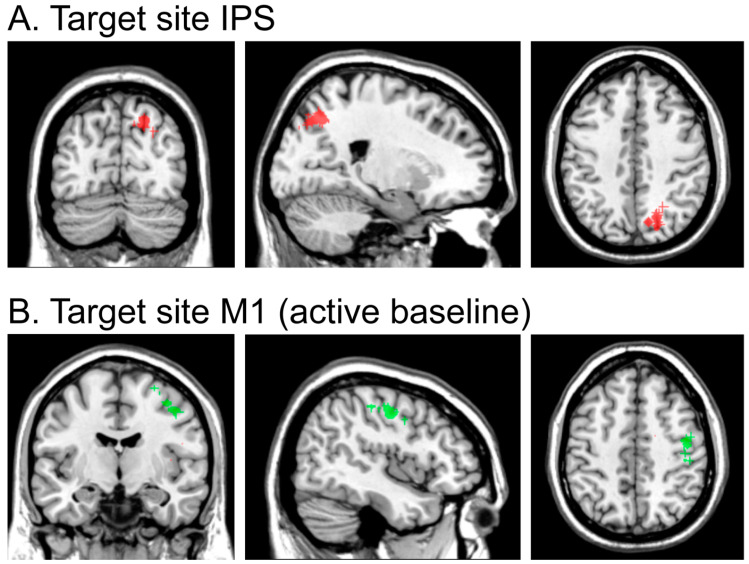
Individual, MRI-guided TMS target sites for all participants. (**A**) Target site in the IPS (shown in red in the figures) with the associated mean MNI coordinates [20, −69, 44]. (**B**) Target site M1 (shown in green in the figures) as used in the active baseline condition, with the associated mean MNI coordinates [41, −10, 51].

Based on the study of Vandenberghe and colleagues [46] who found contralateral effects in a brain-lesioned patient, we chose the target site on the rendered surface of the structural scan on the medial bank of the IPS. To preferentially target more posterior regions analogous to IPS0/1/2 (thought to be particularly important for the allocation of visual spatial attention to the contralateral hemifield; for a review see [23]) and to allow consistent targeting across participants based on neuroanatomical features, we selected the portion of the medial bank of the IPS immediately dorsal to where the middle IPS segment branched off to become what is referred to as the posterior segment of the IPS [46], which usually follows a descending route and becomes the intraoccipital sulcus [47]. This site was, therefore, in the most posterior part of the superior parietal lobe before reaching the occipital lobe. MNI coordinates (see Figure 2A) were similar (within 10 mm) to the coordinates [x = 21, y = −78, and z = 43] reported in [23,48] for a more ventral portion of the medial bank of the posterior IPS.

For the active baseline condition, we searched for the M1 region functionally (Figure 2B). This control site was selected because it is—to our knowledge—(unlike area IPS) not directly related to processes of spatial attentional selection and not associated with deficits that are frequently reported in neglect patients. Moreover, it allowed a similar sensation of being stimulated given that approximate laterality (x = 41) and dorsal-ventral (z = 51) were approximately equivalent to the active site. It should be noted that there was no significant difference in mean stimulation intensity between the IPS and M1 stimulation conditions, *t*(16) = 0.61, *p* = 0.553 (mean intensity = 44.6% and 44.5% maximal stimulator output for IPS and M1 stimulation conditions, respectively).

In the passive baseline condition, the coil was positioned orthogonally to the participant’s scalp such that no effective stimulation could reach the underlying brain tissue. This passive baseline condition was used to control for nonspecific clicking sound and tactile sensation of the TMS pulses [49]. The order of the TMS stimulation conditions was counterbalanced across participants.

## 3. Results

Statistical analyses were performed using repeated-measures analyses of variance (ANOVAs) and subsequent post hoc tests (paired-samples *t*-tests with Holm correction for multiple comparisons) with the program R Studio, version 1.4 [50]. Greenhouse–Geisser corrected values are reported when Mauchley’s test of sphericity was significant (*p* < 0.05).

An initial analysis was performed to estimate the overall level of guessing, by performing a repeated-measures ANOVA on catch trials (i.e., trials without a target but with varying distractors) with the within-subject factors object configurations (ungrouped, left triangle, right triangle, diamond) and TMS stimulations (IPS, M1, no rTMS). The results showed that participants’ performance on trials without a target was very accurate overall (79.6%), thus meeting the intended criteria of 80% accuracy in overall task performance (e.g., as established in a previous pilot experiment, see methods). The ANOVA did not reveal any significant main effects or interactions (all *F*s < 1.78, all *p*s > 0.05). The catch-trial accuracies, therefore, show that participants were able to perform the task without relying too much on guessing responses.

Next, we compared the various types of target in an overall repeated-measures ANOVA on the detection accuracies (but now excluding the catch trial responses) with the factors object configuration (ungrouped, left triangle, right triangle, or diamond), target (unilateral left, unilateral right, bilateral), and TMS stimulation (IPS, M1, no rTMS). This analysis revealed a significant main effect of object configuration, *F*(1.95, 31.20) = 3.53, *p* = 0.042, *η*^2^ = 0.01, showing somewhat higher accuracies in ungrouped (91.7%) than in diamond (89.5%), left triangle (88.6%), and right triangle (87.9%) configurations, alongside with a highly significant 2-way interaction of object configuration by target, *F*(3.72, 59.52) = 11.03, *p* < 0.001, *η*^2^ = 0.06. There were no other significant main or interaction effects in this overall ANOVA (all *p*s > 0.05; see the Supplementary Figure S2 for an overview), thus also showing that the various TMS stimulation conditions did not influence performance.

**Figure 3:**
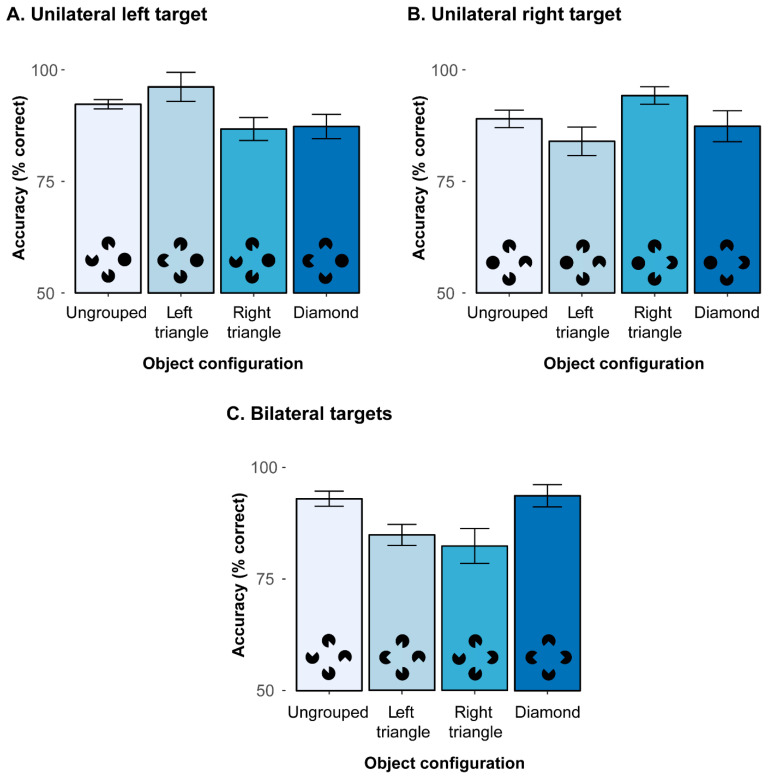
Mean percentages of correct detections (with associated within-subject 95% confidence intervals) as a function of object configuration (ungrouped, left triangle, right triangle, diamond) for (**A**) unilateral left, (**B**) unilateral right, and (**C**) bilateral targets.

To decompose the significant 2-way interaction, additional analyses were performed to compare the various object configurations, separately for the three different types of target (unilateral left, unilateral right, bilateral). First, for unilateral left targets (mean correct detections: 90.6%), there was a significant main effect of object configuration, *F*(1.92, 30.72) = 8.93, *p* < 0.001, *η*^2^ = 0.07 (see Figure 3A). Holm post hoc tests revealed detection accuracies to be (marginally) higher with left triangle configurations (96.2%) than ungrouped configurations, *t*(16) = 2.37, *p* = 0.063, as well as right triangle and diamond configurations, *t*(16)s > 3.24, all *p*s < 0.021. Accuracies for ungrouped configurations were also higher (92.3%) compared to right triangle (86.8%) and diamond (87.3%) configurations, *t*(16)s > 2.93, all *p*s < 0.029. Detection accuracies between right triangle and diamond configurations were comparable, *t*(16) = 0.28, *p* = 0.786. This pattern of results indicates that the emergence of a salient triangle in the left hemifield substantially facilitates left sided, unilateral target detection.

Next, a comparable pattern was also revealed with unilateral right targets (see Figure 3B; mean correct detections: 88.7%), where a comparable ANOVA also resulted in a significant main effect, *F*(2.01, 32.16) = 7.03, *p* = 0.003, *η*^2^ = 0.09. Detection accuracies were significantly higher with right triangle configurations (94.2%) as compared to all other configurations, *t*(16)s > 3.26, all *p*s < 0.019. Moreover, the ungrouped configuration was again somewhat higher in accuracy (89.0%) than the left triangle configuration (84.0%), *t*(16) = 2.70, *p* = 0.047. All other comparisons showed no significant difference (diamond configuration: 87.4%), all *t*(16)s < 2.70, all *p*s > 0.05. Thus, this result pattern for unilateral right targets mirrors the results for the unilateral left targets and once again demonstrates that a salient object configuration in the target hemifield can substantially enhance (unilateral) detection accuracies.

Finally, for bilateral targets (mean correct detections: 88.7%), the main effect of configuration was also significant, *F*(1.74, 27.84) = 11.15, *p* < 0.001, *η*^2^ = 0.08 (see Figure 3C). Accuracies were higher in ungrouped (92.9%) and diamond configurations (93.7%) as compared to the left triangle (84.9%) and right triangle (82.4%) configurations, all *t*(16)s > 3.68, all *p*s < 0.006. Moreover, both ungrouped and diamond configurations and left and right triangle configurations were comparable to each other, *t*(16)s < 0.92, *p*s > 0.739. This shows that the detection of the bilateral targets was hampered whenever a non-integrated but task-relevant target was presented simultaneously with a target embedded in a salient triangle Kanizsa figure in the other hemifield. Compared to the two search displays with a triangle configuration, the ungrouped and diamond configurations resulted in higher accuracies, which possibly resulted from attention being spread more equally across the whole display.

Together, these results show that salient object groupings modulate attentional selection: When the target(s) coincide with the grouped structure, detection performance is improved, while performance is conversely impaired when the salient grouping does not comprise all task-relevant targets. In this latter case, the salient grouping presumably at- tracts attentional resources that are then missing to process the target in the non-salient parts of the display. This overall pattern of results essentially corresponds to the findings reported by [9] in neglect patients, albeit not being confined to one hemifield contralateral to the stimulation site. That is, the concurrent TMS stimulation in area IPS did not yield any significant effects. However, as discussed above, parietal TMS stimulation might not necessarily lead to impaired attentional processing but could also result in an up-modulation of processing, which might then lead to an improvement in performance potentially due to different brain plasticity and, thus, interindividual variability (see introductory section). These opposing effects of TMS might, thus, cancel each other to some extent across different observers and were consequently further examined in a series of follow-up analyses that were performed in an exploratory, post hoc manner after having performed the main analyses as reported above. To this end, we calculated the mean performance across all bilateral trials per participant in the M1 stimulation condition (active baseline) and subtracted it from the mean performance across bilateral trials in the IPS stimulation condition. Out of the complete sample of 17 participants, a subgroup of N = 7 participants showed an overall (stimulus-unspecific) reduction in bilateral detection accuracy (of 7.14%) in the IPS, as compared to the M1 stimulation condition (“IPS-cost” subgroup). A second subgroup of the remaining N = 10 participants conversely revealed an overall benefit in performance (of 7.16%) in detecting bilateral targets in the IPS as compared to the M1 TMS stimulation condition, irrespective of the presented stimulus configuration (“IPS-benefit” subgroup).

The specific variations in performance of these two subgroups were subsequently analyzed in a series of comparisons. It should be noted that, for these analyses, we merged the data from (i) the “left triangle” and the “right triangle” configurations into a single “triangle” condition, and we also combined (ii) unilateral left and right targets to a single “unilateral” target condition. The data were combined in order to increase the number of observations per condition and because the above reported analyses already revealed comparable and “symmetric” effect patterns (e.g., comparable benefits in detecting the unilateral targets in both left and right triangle conditions). However, it should nevertheless be noted that the samples in these two subgroups were rather small, thus leaving only relatively few observations per condition. The results from these exploratory analyses should, thus, be interpreted with caution. In a first step, a mixed 3-way ANOVA with the between-subject factor subgroup (IPS-cost, IPS-benefit), and the within-subject factors target (unilateral, bilateral) and configuration (ungrouped, triangle, diamond) was computed for the no rTMS stimulation condition in order to explore the possibility that the two subgroups already differed without applying any TMS stimulation. This analysis yielded no significant main effects or interactions, including the factor subgroup, all *F*s > 0.17, *p*s < 0.05, thus showing that the two groups were per se comparable, and the different result patterns, thus, must have emerged from the TMS stimulations.

**Figure 4:**
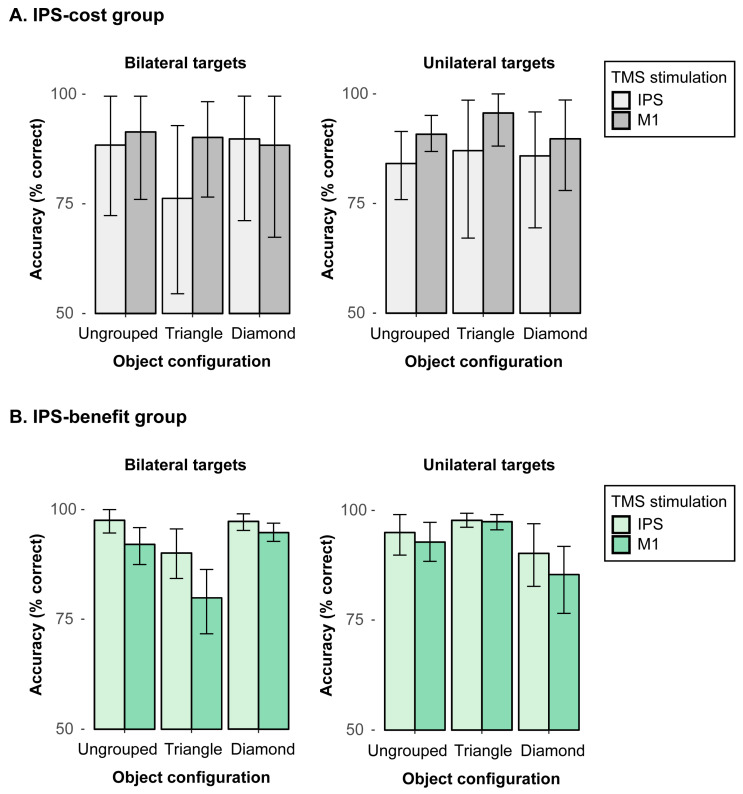
Mean percentages of correct detections (and associated 95% confidence intervals) as a function of object configuration (ungrouped, triangle, diamond) in the IPS and M1 TMS stimulation conditions. The results are depicted for the “IPS-cost” (**A**) and “IPS-benefit” (**B**) subgroups for bilateral target displays (left panels) and for unilateral target displays (right panels).

Next, performance in the “IPS-cost” subgroup was analyzed with a 2-way repeated-measures ANOVA. We found a significant interaction between object configuration (ungrouped, triangle, diamond) and TMS stimulation (IPS, M1) in bilateral targets, *F*(2, 12) = 5.34, *p* = 0.022, *η*^2^ = 0.02 (see Figure 4A, left). In triangle configurations, the mean accuracy was reduced by 13.9% with IPS stimulation (76.2%) as compared to the M1 stimulation (90.1%), *t*(6) = −2.41, *p* = 0.026, whereas there was no significant difference (of 3% and 1.5%) across the TMS stimulation conditions in ungrouped or diamond configurations, respectively, all *t*s (6) < 1.11, *p*s > 0.05 (one-tailed). This pattern shows the IPS stimulation had a rather specific cost of processing bilateral targets, which becomes particularly evident in triangle configurations. That is, the participants in the “TMS-cost” subgroup tended to miss one of the bilateral targets when the display configuration was biased, thus revealing one salient target (i.e., in the triangle) and a second, less salient target item. By contrast, for unilateral targets (see Figure 4A, right), the results showed no significant main or interaction effects, all *F*s < 2.27, *ps* > 0.05.

We then analyzed performance in the “IPS-benefit” subgroup, that is, in those individuals that benefited overall from the IPS stimulation (relative to M1 stimulation). A repeated-measures ANOVA of the mean detection accuracies for bilateral targets with the factors object configuration (ungrouped, triangle, diamond) and TMS stimulation (IPS, M1) showed a significant main effect of object configuration, *F*(2, 18) = 10.42, *p* < 0.001, *η*^2^ = 0.31, revealing reduced accuracies for the triangle (84.9%) as compared to ungrouped (94.8%) and diamond (96.1%) configurations, *t*s (9) > 4.00 *p*s < 0.001. The main effect of TMS stimulation was also significant, *F*(1, 9) = 16.86, *p* = 0.003, *η*^2^ = 0.15, with overall higher accuracies for IPS (94.9%) than M1 (88.9%) stimulation. Moreover, the 2-way interaction was also significant, *F*(2, 18) = 3.87, *p* = 0.040, *η*^2^ = 0.04 (see Figure 4B, left), revealing higher detection accuracies for the IPS than M1 stimulation in ungrouped (IPS: 97.6%; M1: 92.1%) and triangle configurations (IPS: 90.1%; M1: 79.9%), all *t*s (9) < 2.46, *p*s < 0.036, as opposed to no reliable difference with diamond configurations where performance was overall close to ceiling and, thus, only showed a marginal benefit with IPS stimulation relative to the M1 stimulation (IPS: 97.3%; M1: 94.8%), *t*(9) = 2.06, *p* = 0.071 (one-tailed). In addition, the results for unilateral targets only showed a significant main effect of object configuration, *F*(2, 18) = 7.16, *p* = 0.005, *η*^2^ = 0.18 (see Figure 4B, right), showing reduced accuracies for the (incomplete) diamond (87.8%) as compared to ungrouped (93.9%) and triangle (97.6%) configurations, all *t*s (9) > 2.81 *p*s < 0.011. There were no further main or interaction effects that involved the factor TMS stimulation, all *F*s < 4.21, *p*s > 0.05. This pattern shows that for the IPS-benefit subgroup, IPS stimulation improved the detection accuracies of the bilateral target displays in particular in ungrouped and triangle configurations, suggesting that IPS stimulation—in this subgroup—enhanced the spreading of attention across both hemifields.

While performance in the IPS-cost and IPS-benefit subgroups did not differ in the passive, no rTMS baseline stimulation condition (see analysis above), the M1 stimulation might nevertheless have affected performance even though it was meant to serve as an active baseline/control condition. In order to test a potential difference between the passive and active baselines, a final analysis aimed to compare the M1 stimulation condition to the no rTMS stimulation condition. To this end, a series of 2-way repeated-measures ANOVAs with the factors object configuration (ungrouped, triangle, diamond) and TMS stimulation (M1, no rTMS) were performed in the IPs-cost and IPS-benefit subgroups, separately for bilateral and unilateral targets. First, with bilateral targets, the analyses of both subgroups only revealed the main effects of object configuration, *F*s > 5.89, *p*s < 0.02, *η*^2^s < 0.37, but no main or interaction effects that included the factor TMS stimulation, all *F*s < 1.08, *p*s > 0.35. In both the IPS-cost and IPS-benefit subgroups, the detection of bilateral targets showed reduced accuracies in triangle configurations relative to the ungrouped (by 11.1% and 8.8%, respectively) and diamond configurations (by 12.3% and 9.3%, respectively). In addition, with unilateral targets, the identical analyses again yielded main effects of object configuration, *F*s > 5.20, *p*s < 0.03, *η*^2^s < 0.33, but also no effects that included the factor TMS stimulation, all *F*s < 2.59, *p*s > 0.15. With unilateral targets, detection accuracies were enhanced in both the IPS-cost and IPS-benefit subgroups when presented with triangle configurations relative to both the ungrouped (by 4.7% and 4.2%, respectively) and diamond configurations (by 4.7% and 9.1%, respectively). Together, these results, thus, mirror the benefits and costs for the triangle configuration—a pattern that was already described above in the main analysis. However, importantly, these findings also show that there were no TMS-specific effects, thus indicating that the active M1-stimulation baseline did not differ from the passive no-rTMS baseline condition.

## 4. Discussion

The present study investigated whether the posterior parietal cortex mediates the attentional selection of target items and the concurrent organization of the display layout according to perceptual grouping mechanisms. To this end, a sample of healthy participants was stimulated with rTMS over the medial bank of the IPS (as compared to an active, M1, and a passive, no-rTMS, control condition), while performing a target detection task with briefly presented (and subsequently masked) visual search items, which allowed us to probe object integration processes in the left and right visual hemifields. The task required participants to detect lateral targets, which were embedded into different variants of groupings such that individual parts could be integrated into coherent Kanizsa-type illusory objects within the left, the right, or across both visual hemifields.

The results showed that the detection of unilateral targets was enhanced in accuracy when the individual items in the display could be grouped together to form an illusory triangle configuration (that also embedded the target). This result is in line with previous studies who found that salient object groupings tend to capture attention (see, e.g., [51,52,53,54]).

Interestingly, the very same salient triangle configurations within a given hemifield resulted in poorer search performance when there were bilateral, as opposed to unilateral, targets (83.6% vs. 95.2%, *t*s (16) > 5.46, *p*s < 0.001). That is, participants appeared to have missed the non-integrated target when it appeared together with a target embedded in the salient triangle in the other hemifield, suggesting that attention is biased towards the salient grouped structure. By contrast, no comparable reduction in performance was evident for ungrouped and diamond configurations, presumably, because in these configurations, attention was not biased towards one side and could, therefore, spread equally across the whole display. This pattern is largely comparable to the neglect patient’s results as reported in [9]: when attention is currently engaged in one half of the display, other objects are likely to be missed. However, if attention is available, then grouping can increase the conspicuity of a given target, thereby enhancing search efficiency and improving its detectability [54,55,56,57,58] (see also [59]). Importantly, unlike neglect patients, our healthy participants in the current study were able to spread attention equally across both hemifields, suggesting in turn that attention was available to bind fragmentary parts into a coherent whole in the first place, thus triggering the formation of an integrated object (see [9]). This result is also consistent with findings from several masking studies who reported that the integration of separate elements into a coherent whole illusory object is hampered when awareness is unavailable to bind parts to a coherent whole object [35,36,37,38,39].

While the current results are, in general, compatible with the view that object grouping requires attention, TMS stimulation in parietal cortex did not reveal any effect (at least when considering the entire sample of observers). This lack of a modulatory influence might be taken to indicate that the effects of lesions in neglect patients are not directly comparable to the effects observed after TMS stimulation in healthy observers, where effects might also critically depend on the type of stimulation (offline vs. online) and its intensity. Moreover, neglect patients typically show fairly large and variable right-sided lesions in parietal regions, which can extend into temporal, occipital, frontal cortex, and may even propagate into subcortical structures [4,5,60], while the severity of behavioral symptoms may also vary quite substantially across individuals depending on the location and size of the lesion [5,25]. Thus, quite a diverse range of lesions may lead to diverse clinical signs of neglect. Moreover, studies that examined neglect-like symptoms with TMS also varied quite substantially in terms of the specific areas in parietal cortex which were stimulated (e.g., [17,18,20,21,22,61]; see also [24,25], for reviews). A number of these studies targeted the posterior parietal cortex by using an EEG coordinate system, leading to stimulation co-ordinates varying across the angular gyrus, intraparietal sulcus in the superior parietal lobule to the temporoparietal junction [21,61]. Finally, parietal TMS was found to not only inhibit attentional processing, thus leading to costs in performance [17,18,20,21,22,23] (see also [24,25], for reviews) but to also reveal excitatory effects that result in an improvement in performance [27,28,29,30,31] (for a review, see [32]). In light of this large variability in terms of the specific functional localization and the resulting effects upon attention, it may actually not be surprising that our overall analysis revealed no TMS-specific effect. We, therefore, not only analyzed the grand averages across all participants but also focused on an exploratory analysis on individual effect patterns. However, some limitations should be acknowledged. For instance, when interpreting the results, one should consider the post hoc nature of our exploratory (group-wise) TMS analysis, which was partly motivated by the lack of an overall, modulatory influence of the parietal rTMS stimulation upon object completion. The resulting findings should, therefore, be interpreted with caution (given the post hoc nature of our analyses and since the number of observations was limited due to the small sample sizes in the two subgroup analyses). Moreover, future studies with a directed hypothesis would also be necessary to confirm our exploratory findings. In addition, spatial-attentional deficits are also commonly associated with a larger damage of the intraparietal lobule (IPL), which also extends into IPS [62]. Future studies should, therefore, try to not only stimulate IPS but also portions of IPL (e.g., as reported in a clinical study by Gillebert et al. [23]).

That being said, our follow-up analyses resulted in one subgroup (N = 7), who showed an “IPS-cost”, that is, TMS stimulation in the target area IPS had a negative effect on accuracy, as compared to M1 stimulation. This IPS-cost in performance, however, was only evident when observers were presented with bilateral targets (i.e., a condition which would typically result in left-sided extinction behavior in neglect patients), and when being presented with triangle configurations (where the salient triangle would potentially induce an attentional bias). Bilateral detections in these displays showed substantially reduced accuracies subsequent to IPS stimulation (76.2%) as compared to M1 stimulation (90.1%). No comparable difference was observed for the other two types of bilateral configurations (ungrouped, diamond), when comparing the two TMS stimulations (ungrouped—IPS: 88.4%, M1: 91.4%; diamond—IPS: 89.8%, M1: 88.4%). Moreover, no significant differences across TMS stimulation sites were evident in this subgroup when processing unilateral targets (IPS: 85.7%, M1: 92.1%). It, thus, seems that the participants in this subgroup established some extinction-like behavior after right-parietal TMS stimulation: they tended to miss one of two bilateral targets. That is, the typical bias in neglect patients to only attend to single target items (in their attended field) is mirrored in the healthy observer’s performance after IPS stimulation. Moreover, the grouped and, thus, salient target did not seem to be selected at the expense of the other, ungrouped and, thus, less salient target (error probabilities: 4.7% for the non-salient vs. 4.5% for the salient targets). Rather, the targets in these triangle displays were overall more likely to be missed when presented in the left hemifield (error probability: 6.8%) as compared to the right hemifield (2.9%). This shows the right-parietal IPS stimulation in this subgroup indeed resulted in a specific disadvantage of detecting the left-sided target in bilateral displays, which is comparable to the typical extinction behavior seen in neglect patients.

Opposite to this pattern, a second subgroup (N = 10) showed an “IPS-benefit”, that is, in these observers, the IPS stimulation had a positive effect on the detection accuracies, as compared to the stimulation of M1. These participants showed more accurate detections of bilateral targets subsequent to IPS, as compared to M1 stimulation for all three types of configurations (94.9% vs. 88.9%). Thus, in this subgroup, the IPS stimulation seems to have facilitated the spreading of attention across both hemifields, thus improving performance overall. This finding might be related to the idea that neglect patients exhibit some inter-hemispheric imbalance within the attentional network that likely causes their pathological selection bias [5,25]. It has also been shown in this regard that stimulation of the parietal cortex in the unimpaired hemisphere of neglect patients may reverse this cerebral imbalance, which in turn reduces extinction behavior (see, e.g., [27]). In the current subgroup of healthy participants, the IPS stimulation might likewise have “optimized” the cerebral balance in the attentional network (even though healthy observers should tend to reveal balanced cerebral processing in any case). Hence, our IPS stimulation resulted in an overall enhancement of performance with bilateral targets in this subgroup.

## 5. Conclusions

The current study extends previous findings from neglect patients and shows that the parietal cortex plays a crucial role in mediating the attentional selection of integrated objects. The intraparietal sulcus, thus, seems to be involved in processing salient, grouped objects, by allocating attentional resources to to-be grouped items in space [46,48]. The rTMS applied over the intraparietal sulcus may, in this regard, reveal both processing benefits and costs in individual observers. While these bidirectional effects appear to fit well to previous studies (see [32] for a review), the functional causes of these facilitatory and inhibitory effects should, nevertheless, be interpreted with caution—given the post hoc, exploratory nature of our analyses of the individual patterns of performance. Future studies should, therefore, test a directed hypothesis and employ a systematic comparison of observers with IPS-modulated processing benefits and costs to further confirm our current findings.

## Data Availability

The original data of the reported experiment have been made publicly available via the Open Science Framework and can be accessed at https://osf.io/j6dch/ (accessed on 2 May 2025).

## Supplementary Materials

The following supporting information can be downloaded at: https://www.mdpi.com/article/10.3390/brainsci15050483/s1, Figure S1: Examples of the different types of object groupings presented in (A) unilateral left, (B) unilateral right, (C) bilateral, and (D) catch target displays.; Figure S2: Mean percentages of correct detections (and associated within-subject 95% confidence intervals) as a function of object configuration (ungrouped, left triangle, right triangle, diamond) and TMS stimulation (IPS, M1, no rTMS) for bilateral (top), unilateral left (middle) and unilateral right (bottom) target trials.
