# Supplementary material for: Right Parietal rTMS Induces Bidirectional Effects of Selective Attention upon Object Integration
Source: Brain Sci. 2025 May 3;15(5):483. doi: 10.3390/brainsci15050483 (PMC12110648; doi:10.3390/brainsci15050483)
Supplement: Supplementary file 1 [file brainsci-15-00483-s001.zip › brainsci-3511756-supplementary.pdf]

Supplement

Conci, M., Nowack, L., Taylor, P. M. C., Finke, K., & Müller, H. J. Right parietal rTMS induces bidirectional effects of selective attention upon object integration.

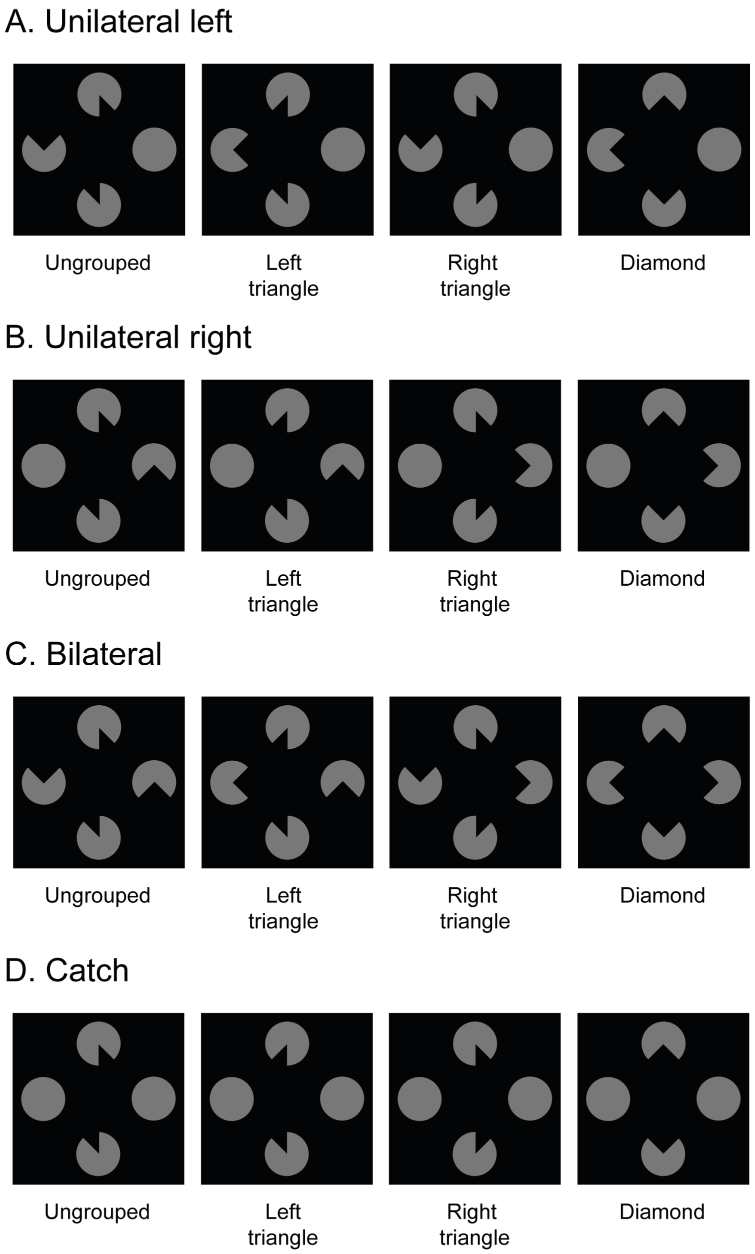

Figure S1. Examples of the different types of object groupings presented in (A) unilateral left, (B) unilateral right, (C) bilateral, and (D) catch target displays.

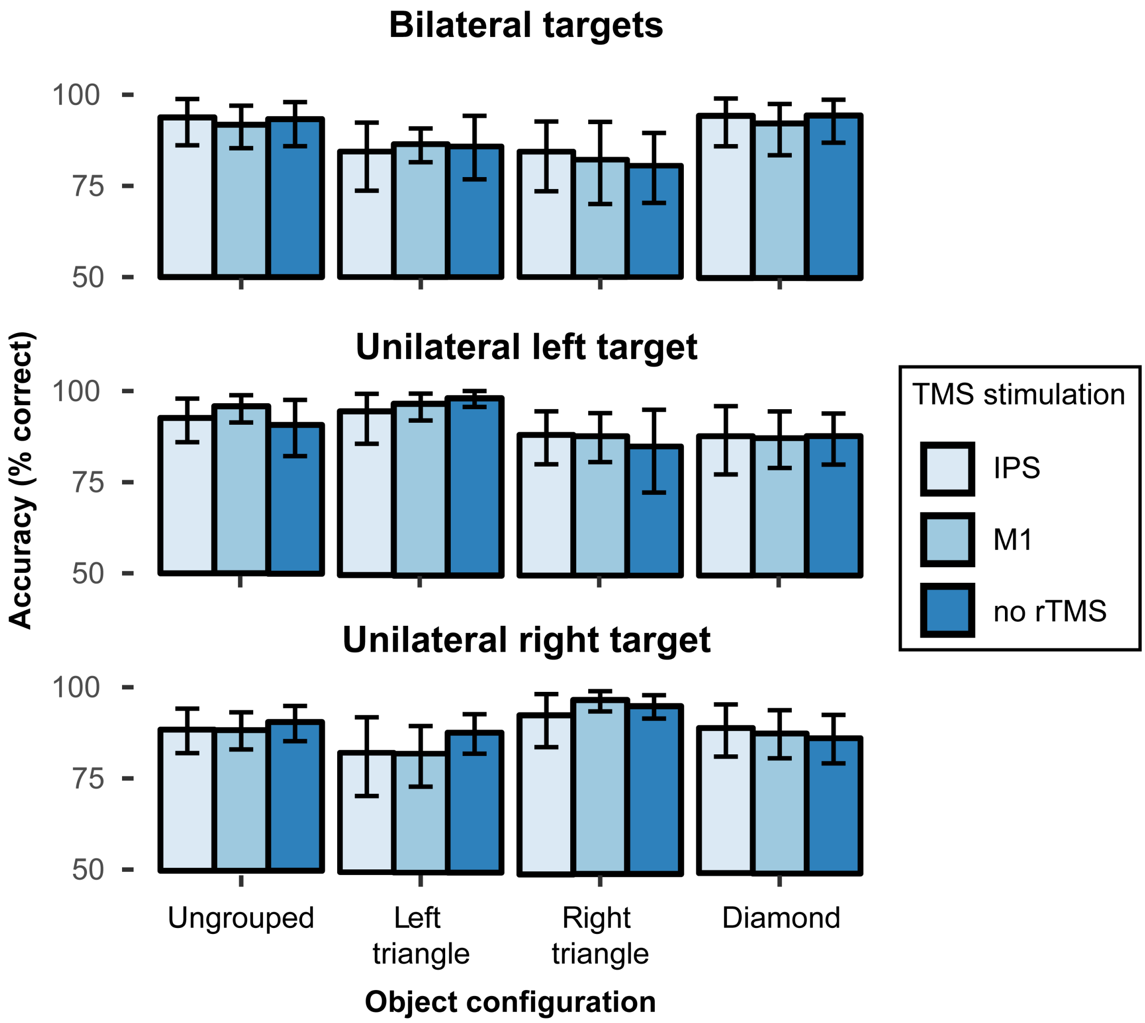

Figure S2. Mean percentages of correct detections (and associated within-subject 95% confidence intervals) as a function of object configuration (ungrouped, left triangle, right triangle, diamond) and TMS stimulation (IPS, M1, no rTMS) for bilateral (top), unilateral left (middle) and unilateral right (bottom) target trials.
